# Adolescent psychological well-being during the COVID-19 lockdown: the role of leisure activities and online peer communication

**DOI:** 10.1007/s12144-022-03679-7

**Published:** 2022-11-04

**Authors:** Anna Di Norcia, Chiara Mascaro, Dora Bianchi, Giordana Szpunar, Eleonora Cannoni

**Affiliations:** Department of Social and Developmental Psychology, “Sapienza” University of Rome, Via dei Marsi 78, 00185 Rome, Italy

**Keywords:** Adolescence, Well-being, Free-time, COVID-19, Online communication

## Abstract

The present study investigated the effects of leisure activities and online peer relationships on the development of psychological difficulties in adolescents during the COVID-19 lockdown in Italy. Data were collected in April and May 2020. The parents of 1,020 Italian adolescents aged 14–18 (51.9% girls) completed questionnaires about the experiences and behaviors of their children before and during the lockdown. A moderation regression analysis was applied to test the research hypotheses. The findings indicated that adolescents who were more active in sports and social activities prior to the COVID-19 pandemic showed greater psychological distress during the lockdown. Moreover, increased reading, game playing, and exercise during the lockdown effectively compensated for the interruption of pre-pandemic sports and social activities, and represented a protective factor for adolescents’ mental health. Finally, increased online contact with peers during the lockdown protected against the development of psychological difficulties, regardless of adolescents’ engagement in sports and social activities prior to the pandemic. The findings provide new and useful information about the role of leisure activities and online contact with peers in protecting against psychological difficulties in adolescents, especially during situations of isolation and social distancing, as in the COVID-19 national lockdown.

## Introduction

The present study aimed at analyzing the role of leisure activities and online relationships with peers in determining or preventing the development of psychological difficulties in adolescents during the COVID-19 lockdown in Italy. Adolescence is a developmental period characterized by the transition from childhood to adulthood, and it is marked by both biological events (e.g., puberty) and changes in social relationships (e.g., with peers and significant adults). All of these changes imply developmental tasks, which aim at the acquisition of a unique adult identity (Havingurst, 1972). Development can be conceived of as a dynamic process of interaction between challenges and resources. The successful resolution of developmental challenges strengthens resources and sustains individual growth. Therefore, growth processes are determined by the interaction between resources, challenges, and the macrosystem of belonging (Hendry & Kloep, 2001).

The COVID-19 pandemic, which affected the entire world in the first months of 2020,was a challenging situation that demanded high psychological resources and resilience. According to Hendry and Kloep (2001), the pandemic was a non-normative historical event that affected everyone and produced significant unexpected changes in the macrosystem. Individuals were unprepared for this event, and forced to reorganize their resources to manage the highly stressful situation. In the months following the COVID-19 outbreak, the Italian government imposed a national lockdown to deal with the health emergency. Despite successfully counteracting the spread of the virus, the lockdown had a detrimental effect on psychological well-being, particularly among adolescents (Caffo et al., 2020; Esposito et al., 2021). As suggested by Singh et al. (2020), during the lockdown, adolescents suffered from an increase in psychological difficulties, due to the impossibility of socializing with peers and the inability to engage in activities that were previously part of their daily routines.

### Free time in adolescence

On the basis of the abovementioned evidence, the present study aimed at analyzing how adolescents organized their free time during the COVID-19 lockdown, hypothesizing that this factor may have impacted their development of psychological difficulties. According to the literature, free time plays a key role in education, learning, and development (Caldwell et al., 2004; Irby & Tolman, 2002). This is particularly true for adolescents, for whom free time is extremely valuable, as it can support their growth by facilitating the resolution of age-specific developmental tasks (Havingurst, 1972; Manetti et al., 2007).

According to Caldwell et al. (2004), free time, if well organized, can contribute to a healthy transition into adulthood. Specifically, the type and quality of leisure activities may support the process of establishing progressive autonomy from parental figures and the development of social roles and adult identity. However, to achieve these benefits, adolescents must not merely “fill” their freetime, but they should instead dedicate it to activities that favor the development of specific competencies (Zill et al., 1995). In this vein, Roberts (1985) described leisure activities as a set of intrinsically satisfying experiences that support socialization and, at the same time, significantly contribute to overall well-being (Gilman, 2001; Miles, 2000).

Many young people spend their free time socializing with friends outside the home, playing sports, engaging in hobbies, or devoting time to themselves (Manetti et al., 2007). Sports, in particular, promote adolescent development through physical and mental challenges, supporting both positive identity development and identification with a peer group; in this respect, they may be considered important for personal growth (Kleiber & Kirshnit, 1991). Indeed, recent studies (Min et al., 2017) have shown that playing sports, in whatever form—and even with minimal participation—can have beneficial psychological effects, particularly on mood.

Adolescents may also spend their free time reading, writing, drawing, or watching movies or television (Gibbons & Stiles, 1997). Pahdy et al. (2020) reported that, in adolescence, well-structured leisure time not only supports positive general development, but it also improves psychological well-being by protecting against problem behaviors and reducing exposure to negative events. Regarding this latter point, a study by Pigaiani et al. (2020) showed that, also during the pandemic, well-organized and structured leisure time increased adolescents’well-being. Specifically, adolescents who spent their time during the lockdown reading, playing board games, engaging in physical activity, and contacting peers reported higher levels of well-being in comparison with peers who did not engage in these activities.

### Online relationships with peers

During adolescence, young people spend relatively more time with peers and relatively less time with parents, in comparison with previous developmental periods (Lam et al., 2014). Furthermore, the influence of peers becomes stronger while parental influence weakens (De Goede et al., 2009; Hadiwijaya et al., 2017). During the lockdown, the amount of time adolescents spent with peers through social media sharply increased, to compensate for the lack of face-to-face contact and the potentially harmful effects of forced isolation (Orben et al., 2020).Therefore, digital devices played a significant role in adolescents’ leisure activities (Hamilton et al., 2020).While it is conceivable that, during the lockdown, adolescents could no longer recoup some of the benefits associated with leisure time spent physically together with peers (Orben et al., 2020), studies suggest that interacting socially with peers through digital devices may have still provided them with important developmental opportunities. The characteristics and qualities of face-to-face interaction are also present in online interactions (Yau & Reich, 2017). Thus, even in the online dimension, adolescents can experience peer relationships, experiment with their identity, and develop relational skills that will be important during adulthood (Subrahmanyam & Greenfield, 2008; Valkenburg & Peter, 2011). Accordingly, for adolescents, time spent with friends (both online and offline) is an important factor (Bukowski et al., 1994) in psychosocial adjustment.

Continuous social contact with peers during adolescence is beneficial for social-emotional functioning at several levels: it sustains good mental health, improves academic performance, and promotes overall positive adjustment (Orben et al., 2020). Contact with peers also provides an important context for identity development (Dahl et al., 2018). Research has broadly shown that online contact with peers may have several benefits. Specifically, during the pandemic, online interaction with peers was found to protect against the negative consequences of physical isolation (Lin et al., 2020; Orben et al., 2020). Beyond the pandemic, it has also been shown to promote adolescents’ psychosocial well-being by facilitating feelings of emotional support and social belonging (Allen et al., 2014). Moreover, online interactions with peers may help adolescents interact with different groups of people, thereby facilitating their access to social support in times of difficulty (Anderson & Jiang, 2018).

During the COVID-19 pandemic, many adolescents used digital devices to cope with the difficulties of the situation and to mitigate feelings of isolation and loneliness (Cauberghe et al., 2021). From this perspective, digital devices and social media may have provided a psychological lifeline for adolescents during the lockdown, allowing them to maintain relationships despite the forced social isolation (Ellis et al., 2020; Magis-Weinberg et al., 2020). Several studies have highlighted that online contact with peers during the COVID-19 lockdown was beneficial for adolescents by, for instance, reducing feelings of loneliness (Magis-Weinberg et al., 2020). Indeed, interactions through digital devices may have protected young people from developing internalizing symptoms during this difficult historical period (Bernasco et al., 2021; Burke et al., 2010; Ellison et al., 2014). However, other studies (Beullens et al., 2011; Henderson, 2017; Lajunen et al., 2009) have analyzed the possible negative outcomes of leisure time spent with peers without parental control, which include aggressive, violent, and antisocial behavior. The same consequences may emerge for online interaction with peers without parental supervision, and such consequences may impair adolescents’ ability to interact with others face-to-face (Orben et al., 2020).

The present study focused on the possible role of peer-to-peer interactions mediated by digital devices and other leisure activities in protecting adolescents from psychological difficulties during the COVID-19 lockdown. Clark (2018) proposed an interesting distinction between active and passive usage of digital devices: With active usage, digital devices are employed to communicate and interact with peers; such usage tends to have a positive effect on psychological well-being (Burke et al., 2010) and helps to maintain personal relationships (Ellison et al., 2014). With passive usage, in contrast, digital devices are employed to simply scroll through social feeds; such usage tends to negatively impact well-being by increasing social comparison and envy (Verduyn et al., 2017).

### Aims and hypotheses

In light of the above mentioned evidence, the present study aimed at investigating the effects of various leisure activities (i.e., pre-lockdown sports and social activities, interaction with peers online, reading, game playing, physical exercise) on the development of psychological difficulties in Italian adolescents during the COVID-19 lockdown. Over the past 2 years, research has provided consistent evidence of an upsurge in psychological distress among adolescents as a result of the lockdown (Esposito et al., 2021; Singh et al., 2020). Some studies have explored the role of leisure activities in this trend (Ettekal & Agans, 2020; Schmidt et al., 2020). However, the novelty of the present study consists in its attention to pre-pandemic lifestyles, in order to evaluate changes in daily habits and the impact of these changes on mental well-being.

Specifically, the study investigated the following research hypotheses:

(H1) Adolescents who most frequently engaged in sports and social activities prior to the lockdown would be at greater risk of experiencing psychological difficulties during the lockdown, as a result of the radical change in their routines. In this perspective, the loss of pre-pandemic sports and social activities was expected to be a risk factor.

(H2) Increased online contact with peers and increased reading, game playing, and physical exercise during the lockdown would reduce psychological difficulties, acting as independent protective factors.

(H3) Increased online contact with peers and increased reading, game playing, and physical exercise would moderate the expected relationship between pre-pandemic sports and social activities and psychological difficulties during the lockdown, counterbalancing the risk of the sudden change in lifestyle.

## Method

### Participants and procedure

The study data came from a larger research project on the psychological well-being of Italian adolescents during the first national lockdown due to COVID-19 pandemic. Data were gathered in April and May 2020, while the Italian population was home confined. The research questionnaires were completed by parents, who reported on the experiences and behaviors of their children both before and during the lockdown. Parents were recruited online via a snowball sampling method, and the link to the survey was disseminated on social networking sites. A preliminary informed consent ensured the voluntariness and anonymity of parents’ participation. Questionnaires were completed in 10 minutes, on average.

Initially, questionnaire data was provided for 1,067 adolescents in the target age range; however, 43 questionnaires were removed due to incompletion, and 4 were removed because the target adolescents were not currently living in Italy, resulting in a response rate of 95.6%. The final sample referred to 1,020 Italian adolescents aged 14–18 years (*M*_*age*_ = 15.57, *SD*_*age*_ = 1.32; 51.9% girls). Most adolescents (75.4%) lived in central Italy, while 19% were based in the north and 5.6% in the south. Regarding education, 83.2% of the adolescents were attending secondary school and 16.8% were attending middle school. The research and study procedure were approved by the ethics committee of the Department of Social and Developmental Psychology of Sapienza University of Rome [*blinded for peer review*].

### Measures

#### Individual information

Parents reported the gender (0 = girl; 1 = boy) and age of the adolescent for whom they were completing the questionnaire.

#### Sports and social activities before the lockdown

Three items evaluated the frequency with which adolescents engaged in particular sports and social activities in a typical week prior to the COVID-19 lockdown: (1) organized or team sports (e.g., basketball, volleyball, dance); (2) other organized group activities (e.g., girl/boy scouts, drama class); and (3) socializing with peers (e.g., meeting friends at home, going out with friends). These items referred to in-person group experiences, which were strictly forbidden during the lockdown. For this reason, the variable was framed as a retrospective assessment of pre-pandemic habits, without a corresponding measure for the pandemic period. Answers were rated on a five-point Likert-type scale, as follows: 0 (*never*), 1 (*rarely*), 2 (*once a week*), 3 (*two or more times a week*), and 4 (*daily*). A total mean score was computed to evaluate the overall frequency of organized sports and social activities before the lockdown.

#### Change in leisure activities (reading, game playing, physical exercise) during the lockdown, relative to the previous period

Three items assessed the frequency with which adolescents engaged in particular leisure activities (with the exception of online activity) in a typical day: (1) reading for pleasure (e.g., novels, comics); (2) playing games (e.g., board games, free play); and (3) practicing physical exercise (e.g., running, lifting weights). Answers were rated on a four-point response scale, as follows: 0 (*not at all*), 1 (*less than 1 hour*), 2 (*approximately1 hour*), and 3 (*2 or more hours*). These activities could take place during the lockdown, as long as they were practiced individually or with cohabitants. Thus, the items were framed in two versions, referring to the pre-lockdown period (version 1) and the current lockdown period (version 2). Mean total scores were obtained for each time period, and a differential score was computed for each participant, to measure the change in the frequency of daily leisure activities during the lockdown, in comparison with the previous period. This differential score ranged from − 3 to 3, with higher scores indicating an increase and lower scores indicating a decrease in the frequency of leisure activities during the lockdown, in comparison with the previous period.

#### Change in online peer communication during the lockdown, relative to the previous period

One item assessed the frequency of online communication with peers during the lockdown, in comparison with the previous period: “Please evaluate the change in the amount of time your child spends online connected with peers (e.g., in video calls, playing video games, etc.; excluding video lessons), in comparison to the pre-lockdown period.” Answers were rated on a three-point response scale, as follows: 0 (*less than before*), 1 (*as much as before*), and 2 (*more than before*). Higher scores indicated an increase, while lower scores indicated a decrease in the amount of online communication with peers during the lockdown, in comparison with the previous period.

#### Psychological difficulties before and during the lockdown

Eight items were created ad hoc to investigate specific problem behaviors in adolescents, as perceived by parents: (1) difficulty staying still; (2) difficulty concentrating; (3) nervousness and irritability; (4) a tendency to cry for no reason; (5) difficulty falling asleep; (6) restlessness during sleep, with nocturnal awakenings; (7) food refusal; and (8) excessive searching for food. Answers were rated on a three-point response scale, as follows: 0 (*never*), 1 (*sometimes*), and 2 (*often*). Items were framed in two versions, referring to the frequency of problem behaviors in the pre-lockdown period (version 1) and during the current lockdown (version 2). Both versions achieved acceptable and good reliability, respectively (Cronbach’s alpha of .60 in version 1, and of .71 in version 2).

### Data analysis

Data analysis was performed using the statistical program SPSS, version 24.0. Descriptive statistics and bivariate Pearson’s correlations were computed for the study variables. The percentage frequencies of all study variables were reported. When the frequencies of both time points (i.e., prior to and during the lockdown) were available, paired-sample *t-*test comparisons were also applied. A moderation regression analysis was then conducted, entering psychological difficulties during the lockdown as the criterion variable. Following the suggestion of Cohen et al. (2002), the independent predictors were preliminarily centered on their grand mean (with the exception of gender, which was dummy coded). Subsequently, two interaction terms were computed in line with the hypotheses: pre-pandemic sports and social activities * change in leisure activities; and pre-pandemic sports and social activities * change in online peer communication. Finally, the moderation model was tested in different steps: in step 1, gender, age, and psychological difficulties prior to the lockdown were entered as control variables; in step 2, pre-pandemic sports and social activities, change in leisure activities, and change in online peer communication were regressed on the criterion; and in step 3, the two interaction terms were added to the regression equation. Since a significant interaction was found, a slope analysis was also conducted to interpret its direction: the predicted values of psychological difficulties during the lockdown were plotted as a function of pre-pandemic sports and social activities, for high (1 *SD* above the mean) and low (1 *SD* below the mean) levels of the moderator (Aiken & West, 1991).

## Results

### Descriptive results

Preliminary analyses of skewness and kurtosis ascertained the normal distribution of variables in the sample (± 2; Tabachnick & Fidell, 2013). Regarding pre-pandemic sports and social activities, 93.9% of adolescents were involved in organized/team sports prior to the pandemic: 25.6% of the sample engaged in these activities weekly or less, 60.3% engaged in them at least twice per week, and 7.6% engaged in them daily.

As regards leisure activities at the investigated time points, parents reported that 34.8% of adolescents read for pleasure (*item 1*) daily (i.e., for at least 1–2 h at a time) prior to the pandemic, in comparison to 39.6% during the lockdown. Overall, this variable showed significant growth during the lockdown, in comparison with the previous period, *Student t* (1019) = 4.30, *p* < .001. Additionally, parents reported that 31.1% of adolescents played games (*item 2*) daily (i.e., for at least 1–2 h at a time) prior to the pandemic, in comparison to 32.8% during the lockdown. On this variable, no significant difference emerged between the two time points, *Student t* (1019) = 1.82, *p* = .07. Finally, parents reported that 74.4% of adolescents engaged in daily exercise (*item 3*) (i.e., for at least 1–2 h at a time) prior to the pandemic, in comparison to 43.4% during the lockdown. This variable showed a significant decrease during the lockdown, *Student t* (1019) = -21.75, *p* < .001. When the overall frequency of leisure activities (mean score of items 1, 2, and 3) was investigated, a significant reduction was found during the lockdown, in comparison with the pre-pandemic period, *Student t* (1019) = -0.69, *p* < .001.

As regards online communication with peers, parents reported that most adolescents (78.0%) increased the amount of daily time they spent communicating with peers online during the lockdown, in comparison with the previous period. Conversely, 15.6% did not vary in this respect, and 6.4% reduced the daily time they spent communicating online with peers during the lockdown. Regarding psychological difficulties, according to parent reports, only 4.0% of adolescents showed a moderate or high level of psychological difficulties (mean scores of 1 or more) prior to the pandemic, while this percentage increased to 18.0% during the lockdown. Overall, the reported psychological difficulties were significantly higher during the lockdown, in comparison with the pre-pandemic period, *Student t* (1019) = 19.23, *p* < .001. Table 1 reports the means and standard deviations for all variables. Table 2 presents the bivariate Pearson’s correlations and descriptive statistics for the main study variables.

**Table 1:** Descriptive Statistics of the Variables Investigated Before and/or During the COVID-19 Lockdown

	Range	Pre-lockdown	During the lockdown		Changeacross time points
		*M* (*SD*)	*M* (*SD*)	*Student t*	*M* (*SD*)
1. Pre-pandemic sports and social activities	0–4	2.13 (0.63)	--	--	--
2. Reading (*item 1*)	0–3	1.15 (0.92)	1.24 (0.97)	*t* (1019) = 4.30^***^	--
3. Playing games (*item 2*)	0–3	1.03 (0.95)	1.08 (1.02)	*t* (1019) = 1.82	--
4. Exercising (*item 3*)	0–3	2.02 (0.92)	1.31 (0.90)	*t* (1019) = -21.75^***^	--
5. Leisure activities (*items 1, 2, 3*)	0–3	1.40 (0.59)	1.21 (0.61)	*t* (1019) = -10.69^***^	-0.19 (0.57)
5. Change in online peer communication	0–2	--	--	--	1.72 (0.57)
6. Psychological difficulties	0–2	1.38 (0.40)	1.88 (0.59)	*t* (1019) = 19.23^***^	--

Note. *** p < .001; ** *p <* .01; * *p <* .05

**Table 2:** Descriptive Statistics and Bivariate Pearson’s Correlations on the Main Study Variables

	1	2	3	4	5	6	7	Range	*M* (*SD*)
1. Gender (0 = boys; 1 = girls)	1							--	--
2. Age	− 0.04	1						14–18	15.57 (1.32)
3. Pre-pandemic sports and social activities	0.03	0.01	1					0–4	2.13 (0.63)
4. Change in leisure activities	− 0.13^***^	0.13^***^	− 0.08^**^	1				-3–3	-0.19 (0.57)
5. Change in online peer communication	0.02	0.08^*^	0.04	0.05	1			0–2	1.72 (0.57)
6. Psychological difficulties before the lockdown	− 0.05	− 0.01	− 0.11^***^	− 0.03	− 0.03	1		0–2	1.38 (0.40)
7. Psychological difficulties during the lockdown	− 0.08^*^	− 0.01	0.03	− 0.16^***^	− 0.07^*^	0.60^***^	1	0–2	1.88 (0.59)

Note. *** p < .001; ** *p <* .01; * *p <* .05

### Moderation regression analysis

The assumptions of the multiple regression analyses were preliminarily verified on the study variables, with variance inflation factors falling within acceptable ranges (from 1.00 to 1.04 for the present study). Step 1 was significant and explained 36.3% of the variance in psychological difficulties during the lockdown, with prior psychological difficulties showing a significant and positive effect. Step 2 added a significant 2.5% to the explained variance: prior psychological difficulties and gender were significant covariates and, controlling for these effects, the change in leisure activities and the change in online peer communication also emerged as significant and negative predictors of psychological difficulties during the lockdown. Finally, step 3 added a significant 1% to the explained variance, detecting a significant effect for the interaction term: pre-pandemic sports and social activities * change in leisure activities. Overall, the model explained 40% of the variance in adolescents’ psychological difficulties during the lockdown (see Table 3).

**Table 3:** Moderation Regression Model

Predictor	Psychological difficulties during the lockdown					
	Step 1		Step 2		Step 3	
	*R* ^*2*^	*beta*	*ΔR* ^*2*^	*beta*	*ΔR* ^*2*^	*beta*
	0.36^***^		0.03^***^		0.01^**^	
Gender (0 = girls; 1 = boys)		− 0.05		− 0.07^**^		− 0.06^**^
Age		− 0.001		0.02		0.02
Psychological difficulties before the lockdown		0.60^***^		0.60^***^		0.60^***^
Pre-pandemic sports and social activities				0.09^***^		0.09^***^
Change in leisure activities				− 0.14^***^		− 0.15^***^
Change in online peer communication				− 0.05^*^		− 0.05^*^
Pre-pandemic sports and social activities * change in leisure activities						− 0.09^***^
Pre-pandemic sports and social activities * change in online peer communication						− 0.003
*ΔF*	*F*(3, 1016) = 192.74^***^		*ΔF*(3, 1013) = 18.28^***^		*ΔF*(2, 1011) = 6.38^***^	
Total *R*^2^	0.40^***^					

Note. *** p < .001; ** *p <* .01; * *p <* .05

### Slope analysis

**Fig. 1:**
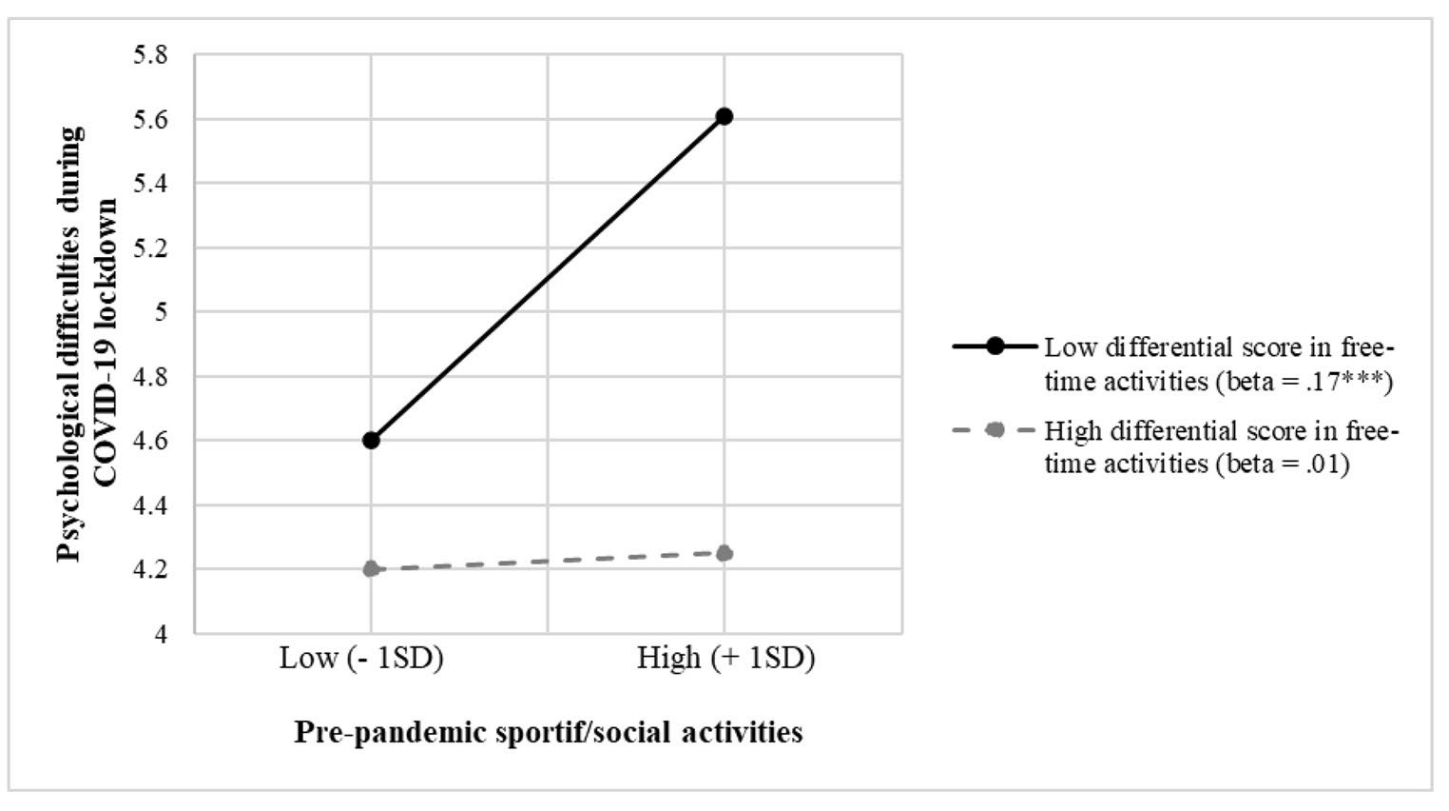
Moderation Effect of Change in Leisure Activities During the Lockdown in the Relationship Between Pre-Pandemic Sports and Social Activities and Psychological Difficulties During the COVID-19 Lockdown Notes: The dashed line represents a non-significant relationship. *** p < .001

In the subsequent slope analysis, the relationship between pre-pandemic sports and social activities and psychological difficulties during the lockdown was plotted at two levels of the moderator (change in leisure activities), while controlling for all model variables. At low levels of the moderator (i.e., when the differential score of leisure activities was low, indicating participants who decreased their leisure activities during the lockdown in comparison to the previous period), the relationship between pre-pandemic sports and social activities and psychological difficulties during the lockdown was positive and significant (*beta* = 0.17, *p* < .001). Conversely, at high levels of the moderator (i.e., when the differential score of leisure activities was high, indicating adolescents who increased their frequency of leisure activities during the lockdown), the same relationship showed a non-significant effect (*beta* = 0.01, *p* = .78). This interaction effect is represented in Fig. 1.

## Discussion

Studies have shown that the COVID-19 lockdown and its associated restrictions had a negative effect on the mental health of children and adolescents (Ezpeleta et al., 2020). In this perspective, the present study aimed at exploring the possible impact of leisure activities and online contact with peers on adolescents’ well-being during the lockdown, taking into account both the type of leisure activity and adolescents’pre-pandemic habits, as reported by parents.

The first hypothesis (H1) was confirmed by the study findings, as the frequency with which adolescents engaged in sports and social activities prior to the lockdown emerged as a positive predictor of psychological difficulties. Specifically, adolescents who were more active in sports and social activities prior to the pandemic showed more psychological difficulties during the lockdown, compared to their less active peers. Thus,the reduction in physical activity and social interaction during lockdown appeared to impair psychological well-being, in line with previous studies showing the negative effect of reduced physical activity and peer relationships during the pandemic (Ezpeleta et al., 2020; Segre et al., 2020). This finding could be explained by evidence that physical exercise promotes physical and psychological health (Bann et al., 2019), and thus a sudden and unexpected restriction of physical activity could lead to a deterioration in psychological functioning.

The present findings suggest that the radical change in daily habits and the deprivation of social and sports activities had more severe consequences for psychological well-being among adolescents who were more active prior to the lockdown. Conversely, those who engaged in fewer activities prior to the lockdown did not experience a major change in lifestyle during the lockdown, and were consequently less affected by the restrictions. In general, children and adolescents are more adaptable than adults; however, when change is sudden, radical, and disruptive of routines, it may give rise to emotional vulnerability and mood instability (Dragun et al., 2021).

Regarding the second hypothesis (H2), the findings showed that online contact with peers protected against the development of psychological difficulties during the lockdown. The lockdown’s abrupt and prolonged interruption of social life (Hamilton et al., 2020) has been shown to have led to a sharp increase in the use of digital devices—not only for educational purposes, but also for recreational and social purposes (Ezpeleta et al., 2020). Thus, digital devices may have represented important tools for coping with the imposed isolation, facilitating the maintenance of social relationships, alleviating distress and concerns about the pandemic (Jiao et al., 2020), and contributing to the healthy regulation of emotional states (Cauberghe et al., 2021; Magson et al., 2021). Moreover, the positive effect of online contact with peers that was found in the present study may be explained by the important role played by peer relationships during adolescence. At this time, social comparison processes are particularly activated, and young people tend to rely heavily on their peers for emotional support, approval, self-esteem, and reassurance (Crone & Fuligni, 2019); Schriber & Guyer, 2016).

On the one hand, the present results suggest that online contact with peers might have mitigated the harmful effects of social isolation during the lockdown, in support of recent research. Many studies have shown that time spent on digital devices during the pandemic represented a positive factor that counteracted social isolation and helped adolescents to maintain contact with peers (Dong et al., 2020; Hamilton et al., 2020; Magson et al., 2021; Orben et al., 2020). On the other hand, the results may also extend beyond the pandemic situation, because research from before the pandemic has also shown that online interactions can promote the well-being of adolescents by allowing them to maintain contact with peers despite physical distance (Burke et al., 2010; Ellison et al., 2014; Subrahmanyam & Greenfield, 2008; Valkenburg & Peter, 2011).

Regarding the second hypothesis (H2), the findings also showed that other leisure activities (i.e., reading, game playing, physical exercise) preserved adolescents’ well-being and protected against the development of psychological difficulties during the lockdown. These results confirm and expand the work of Chen et al. (2020), who showed the positive effects of regular physical activity for adolescents during the lockdown. Specifically, the findings show that positive effects could also be obtained through other leisure activities, including game playing and reading for pleasure.

Finally, regarding the moderation hypothesis (H3), the findings indicated that increased reading, game playing, and physical activities during the lockdown effectively compensated for a lack of pre-pandemic social and sports activities, thereby nullifying the negative effects of this variable on adolescents’ mental well-being. Only for adolescents who did not report increased reading, game playing, and movement during the lockdown, social and sports activities prior to the pandemic were predictive of greater psychological difficulties. As a similar interaction effect was not found for online contact with peers during the lockdown, H3 was only partially confirmed.

These findings may be explained in the light of evidence about the protective role played by structured leisure activities during the lockdown. Such activities have been shown to be constitutive of an adaptive coping strategy, which could support individuals in positively managing the challenges of the pandemic (Pigaiani et al., 2020). The present study also provides new evidence that indoor structured activities (e.g., reading for pleasure, game playing, physical exercise) may have been effective in compensating for the lack of other structured pre-pandemic activities (i.e., organized social and sports activities). While online contact with peers showed an independent positive effect on psychological well-being, it was unable to fully compensate for the lack of face-to-face social and sports experiences. Thus, the results suggest that online interaction with peers may be useful for adolescents’ well-being, but it is unlikely to effectively substitute for face-to-face interactions. Conversely, indoor structured leisure activities may effectively substitute for in-person group activities when necessary, thereby protecting well-being.

## Conclusion

The COVID-19 pandemic posed a significant challenge for adolescents, whose developmental phase is characterized by rapid physical, social, cognitive, and emotional changes, with important implications for health and well-being (Azzopardi et al., 2019). The present study analyzed the impact of the pandemic on the psychological well-being of adolescents, shedding light on the detrimental effects of the sudden interruption of pre-pandemic sports and social activities, and addressing the protective role played by indoor leisure activities and online contact with peers during the lockdown. The findings also provide preliminary evidence of the moderating role played by leisure activities in the hypothesized relationship between pre-pandemic sports and social activities and increased psychological difficulties during the lockdown.

Despite the contribution of these findings to the literature, the study is not exempt from limitations. First, the data collection relied on parent-report surveys, rather than self-report questionnaires. This was due to ethical reasons associated with the lockdown condition: on the one hand, it would have been very difficult to ensure parental consent before involving adolescents in a self-report online and anonymous survey; and on the other hand, it would have been very difficult to guarantee the absence of parental control and other intervening factors (i.e., environmental control) during the administration of the online survey to adolescents. However, it is important to note that parents reported their own perceptions of their children’s psychological well-being. Future multi-informant studies should be implemented to confirm the present findings. Second, the study was based on a retrospective design, due to the specific condition of the unexpected health emergency. Therefore, future longitudinal studies are desirable to confirm the results. Third, the survey did not include a standardized measure for assessing psychological difficulties, because it was designed ad hoc as a short and easy tool, in order to prevent participants’ overload and dropout. Finally, the usual limitations of observational studies apply, in that no causal relationships could be inferred among the study variables.

Notwithstanding these limitations, the results have relevant research and practical implications. Future research should seek to verify the potential maintenance and long-lasting effects of the coping strategies developed during the lockdown. Specifically, research should investigate whether the coping strategies that adolescents implemented during the lockdown remain in their storehouse of potential resources in the post-pandemic period, or whether they are no longer implemented beyond the difficult period of isolation. Additionally, educational and preventive programs targeting adolescents in the post-pandemic period should focus on the protective factors that emerged in the present study, encouraging structured leisure activities such as reading, game playing, and physical exercise, while also favoring online contact with peers, in order to counteract the psychological difficulties associated with the pandemic.

## Data Availability

The data that support the study findings are available from the corresponding author, upon request.
